# A gateway to more productive research on e‐cigarettes? Commentary on a comprehensive framework for evaluating public health impact

**DOI:** 10.1111/add.13449

**Published:** 2016-07-22

**Authors:** Jamie Brown

**Affiliations:** ^1^ Department of Clinical, Educational and Health PsychologyUniversity College LondonLondonUK; ^2^ Cancer Research UK Health Behaviour Research CentreUniversity College LondonWC1E 6BTUK

**Keywords:** E‐cigarettes, harm reduction, modelling, public health, smoking, tobacco

## Abstract

Commentary to: A framework for evaluating the public health impact of e-cigarettes and other vaporized nicotine products

*The widespread use of the comprehensive framework proposed by Levy and colleagues for evaluating the overall public health impact of e‐cigarettes would advance the field. Time–series analysis of frequent cross‐sectional population data on smoking and quitting activities could be invaluable in estimating key parameters for such models*.

E‐cigarettes are controversial among tobacco control and public health experts 1, 2, 3. Part of the reason is that opponents and proponents focus upon different parts of the complicated equation that will determine their overall impact on public health, and there have been few attempts to quantify and integrate all the determinants into an overarching population model 4, 5. Levy and colleagues describe a novel and ambitious framework for evaluating the public health impact of e‐cigarettes 6. A particular attraction is that their framework appears comprehensive and necessarily complex. The authors draw upon systems thinking and decision theory to cover all possible transitions among never, current and former smokers to a variety of final states of nicotine use. In each case, they argue that the overall impact will depend upon how e‐cigarette use influences the long‐term prevalence of each final state compared with the counterfactual situation in which e‐cigarettes do not exist.

This type of framework helpfully draws attention to the possibility of certain scenarios in which e‐cigarettes reduce overall harm, despite the assumption of many opponents that they will increase harm: for example, e‐cigarettes could actually reduce harm to never smokers who would have otherwise initiated long‐term cigarette use. It would be a huge advance if this paper moved the field towards consensus on the key parameters necessary to produce judgements on the overall population impact of e‐cigarettes. Adoption of a common framework and language would make the field more productive, even if researchers continued to disagree on how to interpret different studies when parameterizing and weighting different parts of the model. The positive impact of a ‘Russell Standard’ for evaluating clinical interventions is testament to the importance of common language in the field 7.

Levy and colleagues employ the novel framework to structure a balanced and progressive review of literature that could be used to parameterise the model 6. Greater benefit will arise when using the model to simulate appraisals of overall impact under a variety of different estimates and assumptions. In terms of literature and methods for parameterizing the model, the paper cites trials and cross‐sectional surveys, and notes that interpretation of the latter can be enhanced by matching methods, which have been used to address a variety of thorny issues in tobacco control 8, 9, 10. Finally, their conclusion emphasizes the importance of large‐scale longitudinal studies, such as the US Population Assessment of Tobacco and Health (PATH) survey and the International Tobacco Control (ITC) surveys. The ITC will be particularly important beyond its contribution of longitudinal data by also providing a means of evaluating natural experiments arising from different countries adopting different regulatory approaches 11, 12.

An additional type of data—not explicitly mentioned in their commentary—is likely to prove valuable in this context; namely, frequent cross‐sectional time–series population data on important smoking and quitting activities. Time–series analysis of these population trends would provide direct estimates of both population level associations between variables over time and the associated impact of new policies and regulations, while adjusting for important confounders and seasonal and long‐term trends 13. Associations cannot establish a causal association unequivocally but they can be indicative as, for example, in estimates of the relationships between price and cigarette consumption 14, mass media expenditure and the use of freely available behavioural support 15, changes to national incentives for general practitioners to offer evidence‐based advice and referral behaviour 16 and the introduction of varenicline and use of other cessation medication 17. In a more relevant recent example, we have argued that the growth in the use of e‐cigarettes by English smokers was probably not responsible for the decline in the use of licensed nicotine products 18. In this example, our time–series evidence could be a factor in estimating the counterfactual pathway of current smokers in Levy and colleagues' framework because the number of smokers who would have quit in the absence of e‐cigarettes is not due only to their relative efficacy in cessation, but also whether and how their availability effects other quitting activity.

Importantly, Levy and colleagues acknowledge that critical information for populating the framework will need to be updated continually, and likely only directly applicable to contexts from which that information is derived. Many of the estimated transitions are liable to depend upon a variety of cultural and contextual factors, including the prevailing tobacco control environments.

Tobacco harm reduction from clean nicotine delivery is at least 40 years old 19. The idea is beautifully simple, but this paper by Levy and colleagues underscores the complexity in evaluating the impact of related products. While the current focus is on e‐cigarettes, other novel devices—such as heat‐not‐burn tobacco products—loom that will complicate the modelling 20. In order to minimize the growing number of global avoidable deaths from tobacco, scientists should rely upon clear and common frameworks with explicit parameterization when considering the range of possible public health effects.

## Declaration of interests

J.B. has received unrestricted research grants to study smoking cessation from Pfizer; there are no other financial relationships with any organisations that might have an interest in the submitted work and there are no other relationships or activities that could appear to have influenced the submitted work.

## References

[1] Green S. H. , Bayer R. , Fairchild A. L. Evidence, policy, and e‐cigarettes—will England reframe the debate? N Engl J Med 2016; 374: 1301–3. 27050203 10.1056/NEJMp1601154

[2] McNeill A. , Brose L. S. , Calder R. , Hitchman S. C. , Hajek P. , Mcrobbie H. Ann McNeill and colleagues reply to Martin McKee and Simon Capewell. BMJ 2015; 351: h5010. 10.1136/bmj.h5010 26403170

[3] Nutt D. J. , Phillips L. D. , Balfour D. , Curran H. V. , Dockrell M. , Foulds J. *et al.* E‐cigarettes are less harmful than smoking. Lancet n.d.; 387: 1160–62. 10.1016/S0140-6736(15)00253-6 27025332

[4] Kalkhoran S. , Glantz S. A. Modeling the health effects of expanding e‐cigarette sales in the United States and United Kingdom: a Monte Carlo analysis. JAMA Intern Med 2015; 175: 1671–80. 26322924 10.1001/jamainternmed.2015.4209 PMC4594196

[5] Cobb C. O. , Villanti A. C. , Graham A. L. , Pearson J. L. , Glasser A. M. , Rath J. M. *et al.* Markov modeling to estimate the population impact of emerging tobacco products: a proof‐of‐concept study. Tob Regul Sci 2015; 1: 129–41.

[6] Levy D. , Cummings K. M. , Villanti A. , Niaura R. , Abrams D. B. , Fong G. T. *et al.* A Framework for evaluating the public health impact of e‐cigarettes and other vaporized nicotine products. Addiction 2017; 112: 8–17. 27109256 10.1111/add.13394 PMC5079857

[7] West R. , Hajek P. , Stead L. , Stapleton J. Outcome criteria in smoking cessation trials: proposal for a common standard. Addiction 2005; 100: 299–303. 15733243 10.1111/j.1360-0443.2004.00995.x

[8] Thomas K. H. , Martin R. M. , Davies N. M. , Metcalfe C. , Windmeijer F. , Gunnell D. Smoking cessation treatment and risk of depression, suicide, and self harm in the Clinical Practice Research Datalink: prospective cohort study. BMJ 2013; 347: f5704. 10.1136/bmj.f5704 PMC3805476 24124105

[9] Timberlake D. S. , Huh J. , Lakon C. M. Use of propensity score matching in evaluating smokeless tobacco as a gateway to smoking. Nicotine Tob Res 2009; 11: 455–62. 19307445 10.1093/ntr/ntp008

[10] Beard E. , Aveyard P. , Brown J. , West R. Assessing the association between the use of NRT for smoking reduction and attempts to quit smoking using propensity score matching. Drug Alcohol Depend 2012; 126: 354–61. 22748518 10.1016/j.drugalcdep.2012.05.039

[11] Yong H. H. , Borland R. , Balmford J. , Mcneill A. , Hitchman S. , Driezen P. *et al.* Trends in e‐cigarette awareness, trial, and use under the different regulatory environments of Australia and the United Kingdom. Nicotine Tob Res 2015; 17: 1203–11. 25358657 10.1093/ntr/ntu231 PMC4598793

[12] Gravely S. , Fong G. T. , Cummings K. M. , Yan M. , Quah A. C. K. , Borland R. *et al.* Awareness, trial, and current use of electronic cigarettes in 10 countries: findings from the ITC project. Int J Environ Res Public Health 2014; 11: 11691–704. 25421063 10.3390/ijerph111111691 PMC4245638

[13] Jebb A. T. , Tay L. , Wang W. , Huang Q. Time series analysis for psychological research: examining and forecasting change. Front Psychol 2015; 6: 727. 26106341 10.3389/fpsyg.2015.00727 PMC4460302

[14] Gallus S. , Schiaffino A. , Vecchia C. L. , Townsend J. , Fernandez E. Price and cigarette consumption in Europe. Tob Control 2006; 15: 114–19. 16565459 10.1136/tc.2005.012468 PMC2563577

[15] Langley T. , Szatkowski L. , Lewis S. , Mcneill A. , Gilmore A. B. , Salway R. *et al.* The freeze on mass media campaigns in England: a natural experiment of the impact of tobacco control campaigns on quitting behaviour. Addiction 2014; 109: 995–1002. 24325617 10.1111/add.12448

[16] Szatkowski L. , Aveyard P. Provision of smoking cessation support in UK primary care: impact of the 2012 QOF revision. Br J Gen Pract 2015; 66: e10–5. 10.3399/bjgp15X688117 PMC4684030 26639948

[17] Langley T. E. , Huang Y. , Mcneill A. , Coleman T. , Szatkowski L. , Lewis S. Prescribing of smoking cessation medication in England since the introduction of varenicline. Addiction 2011; 106: 1319–24. 21395894 10.1111/j.1360-0443.2011.03426.x

[18] Beard E. , Brown J. , Mcneill A. , Michie S. , West R. Has growth in electronic cigarette use by smokers been responsible for the decline in use of licensed nicotine products? Findings from repeated cross‐sectional surveys. Thorax 2015; 70: 974–78. 26209508 10.1136/thoraxjnl-2015-206801 PMC4602271

[19] Russell M. A. Low‐tar medium‐nicotine cigarettes: a new approach to safer smoking. BMJ 1976; 1: 1430–33. 953530 10.1136/bmj.1.6023.1430 PMC1640397

[20] Picavet P. , Haziza C. , Lama N. , Weitkunat R. , Ludicke F. Comparison of the pharmacokinetics of nicotine following single and ad libitum use of a tobacco heating system or combustible cigarettes. Nicotine Tob Res 2016; 18: 557–63. 26438645 10.1093/ntr/ntv220 PMC4826489

